# The Association between Pulse Wave Velocity and Cognitive Function: The Sydney Memory and Ageing Study

**DOI:** 10.1371/journal.pone.0061855

**Published:** 2013-04-30

**Authors:** Joel Singer, Julian N. Trollor, John Crawford, Michael F. O’Rourke, Bernhard T. Baune, Henry Brodaty, Katherine Samaras, Nicole A. Kochan, Lesley Campbell, Perminder S. Sachdev, Evelyn Smith

**Affiliations:** 1 Brain and Aging Research Program, School of Psychiatry, University of New South Wales, Sydney, Australia; 2 Department of Developmental Disability Neuropsychiatry, School of Psychiatry, University of New South Wales, Sydney, Australia; 3 St Vincent's Hospital, Darlinghurst, Australia; 4 Victor Chang Cardiac Research Institute, Darlinghurst, Australia; 5 Garvan Institute of Medical Research, Darlinghurst, Australia; 6 Discipline of Psychiatry, School of Medicine, University of Adelaide, Adelaide, Australia; 7 Neuropsychiatric Institute, Prince of Wales Hospital, Randwick, Australia; 8 Dementia Collaborative Research Centre, University of New South Wales, Sydney, Australia; University of Perugia, Italy

## Abstract

**Objectives:**

Pulse wave velocity (PWV) is a measure of arterial stiffness and its increase with ageing has been associated with damage to cerebral microvessels and cognitive impairment. This study examined the relationship between carotid-femoral PWV and specific domains of cognitive function in a non-demented elderly sample.

**Method:**

Data were drawn from the Sydney Memory and Ageing Study, a cohort study of non-demented community-dwelling individuals aged 70–90 years, assessed in successive waves two years apart. In Wave 2, PWV and cognitive function were measured in 319 participants. Linear regression was used to analyse the cross-sectional relationship between arterial stiffness and cognitive function in the whole sample, and separately for men and women. Analysis of covariance was used to assess potential differences in cognition between subjects with PWV measurements in the top and bottom tertiles of the cohort. Covariates were age, education, body mass index, pulse rate, systolic blood pressure, cholesterol, depression, alcohol, smoking, hormone replacement therapy, apolipoprotein E ε4 genotype, use of anti-hypertensive medications, history of stroke, transient ischemic attack, myocardial infarction, angina, diabetes, and also sex for the whole sample analyses.

**Results:**

There was no association between PWV and cognition after Bonferroni correction for multiple testing. When examining this association for males and females separately, an association was found in males, with higher PWV being associated with lower global cognition and memory, however, a significant difference between PWV and cognition between males and females was not found.

**Conclusion:**

A higher level of PWV was not associated with lower cognitive function in the whole sample.

## Introduction

Vascular disease and its related brain pathologies (e.g., stroke, silent brain infarction, subclinical brain injury) are associated with cognitive decline and dementia [1]. Additionally, traditional cardiovascular risk factors such as hypertension, hyperlipidemia and diabetes mellitus are recognized risk factors for Alzheimer's disease [2]. Understanding the mechanisms involved in vascular disease in the elderly is therefore of substantial clinical importance.

One vascular process implicated in cognitive change is arterial stiffening, a hallmark of vascular aging caused by structural change within the aorta and other elastic arteries [3], which is predictive of total and cardiovascular mortality, end-stage renal disease and diabetes mellitus [4]. Increased arterial stiffness adversely affects the brain, with high pulsatile flow damaging cerebral microvessels, leading progressively to edema, hemorrhage and inflammation [3], [5]. As a result, increased arterial stiffness is associated with both cerebral lacunar infarction and cortical brain atrophy [3], [6]. While cross-sectional studies [6], [7], [8], [9], [10], [11], [12], [13] found an association between arterial stiffness and cognitive deficit, longitudinal studies [12], [14], [15], [16], [17] identified arterial stiffness independently contributing to cognitive decline in the elderly.

However, several methodological limitations have been identified [8], [9], [10], [14], [15] such as an over-reliance on imprecise screening rather than diagnostic cognitive measures (e.g., Mini-Mental State Exam (MMSE)) [18]. When detailed cognitive assessment batteries were employed, mixed results regarding the type of cognitive domain affected were reported. While in some studies learning and memory domains were most significantly associated with arterial stiffness [11], [12], [16], others found either executive function [13] or processing speed [17], or two domains simultaneously, such as memory and executive function [7], related to arterial stiffness. The varied results might also be accounted for by the inconsistent use of covariates with some studies controlling only for basic demographic and cardiovascular risk factors [6], [10], [11] while others employed multiple adjustments for medical, psychological, genetic and lifestyle factors [7], [8], [16]. Many studies [6], [8], [9], [10], [11], [12], [13], [14], [15] failed to account for factors such as depression (shown to have an independent effect on cognition [19]), and only two studies controlled for APOE genotype [7], [13], a risk factor for cognitive decline [20]. Finally, several studies lacked rigorous statistical procedures such as Bonferroni correction for multiple analyses [7], [11], [12], [13], [17].

This study investigates the cross-sectional association between arterial stiffness (using carotid-femoral pulse wave velocity (PWV)) and cognition in a large, non-demented community-dwelling elderly population. We hypothesize that there will be a negative association between PWV and global cognition, memory, executive function and speed of information processing as identified in our review of the literature. Since sex differences have been previously reported in the relationship between pulse pressure, another measure of arterial stiffness, and a memory task [16], we hypothesize that this study will detect sex differences in the relationships between PWV and cognition measures, with males displaying a stronger association between increased PWV and decreased cognitive function. In order to examine the impact of a high PWV on cognition, this study also explores whether there are any significant differences in cognitive performance between subjects with PWV measurements in the top and bottom tertiles of the cohort.

## Methods

### Participants

Ethics approval for this study was granted by the University of New South Wales and the South-Eastern Illawarra Area Health Service – Eastern sector (HREC 09382). Written informed consent was obtained from all participants. Participants were drawn from the community-based Sydney Memory and Aging Study (MAS). The study is described in detail elsewhere [21]. Briefly, 1,037 non-demented community dwelling adults aged 70–90 years were recruited through the electoral roll at baseline. Exclusion criteria were: psychosis, intellectual delay, multiple sclerosis or any other medical condition producing incompetence to participate, a MMSE [18] score adjusted for age and education <24 or a diagnosis of dementia [22]. All participants were assessed at baseline (Wave 1 and 889 were assessed again two years later (Wave 2). Of the original sample, at Wave 2 43 were deceased and 105 declined further participation. Of the remaining 741, only 480 at Wave 2 agreed to an additional cardiovascular assessment, which included the measurement of Pulse Wave Velocity. Because of uncertainty of the validity of their neuropsychological test scores, non-English speaking background participants were then excluded from this group (n = 96). A complete data set was available for 319 participants.

### Neuropsychological Measures

A battery of neuropsychological tests was administered by trained psychology graduates. Eleven tests were administered measuring four cognitive domains of memory, processing speed, language, and executive abilities, representing cognitive functions commonly affected by ageing and early dementia. The tests were categorised into domains *a priori,* based on the principal cognitive function they represented according to convention and psychological theory [23]. To assess processing speed, Digit Symbol Coding [24], and Trail Making Test A [25] were used. Memory was measured via Logical Memory Story A (delayed) [26], Rey Auditory Visual Verbal Learning Test (RAVLT) (total learning; trials 1–5, short-term recall; trial 6 and long-term recall; trail 7) [27] and the Benton Visual Retention Test (BVRT) [28]. Animal Naming [29] and the 30-item Boston Naming Test [30],[31] were used to assess Language. Executive Function was assessed using Phonemic Fluency (FAS) [32], Trail Making Test B [25] and the Stroop Test [33]. Visuospatial ability was measured via Block Design [34]. Cognitive domain composite scores were formed by first transforming the component test scores to z-scores based on the means and standard deviations of the English speaking Wave 2 sample, and then averaging the z-scores of the component tests and transforming to normal scores, using Blom's procedure. A composite global cognition score was created by summating the z scores of the domains mentioned.

### PWV

Carotid-Femoral PWV was measured with the SphygmoCor PVx system (AtCor, Sydney, Australia). Participants were placed in a supine position with three ECG monitoring electrodes on their chests to allow ECG synchronisation. A high fidelity tonometer was placed sequentially over the right carotid and femoral pulses and gently pressed down until a steady waveform was achieved. Using ECG synchronisation and the formula CF-PWV = δD/δt, CF-PWV in m/s was calculated. Distance between recording sites (δD) was measured via the surface distance and Time (δt) was measured between the feet of the two wave forms [3].

### Covariates

All analyses of the relationships between arterial stiffness and cognition were performed with the participants' age and years of education as control variables (‘Model 1’). Sex was also included for all whole-sample analyses. Additional covariates were selected based on previous literature indicating significant effects on arterial stiffness, cognitive function or both (‘Model 2’): systolic blood pressure, pulse rate, depression, apolipoprotein E (*APOE*), Body Mass Index, stroke, transient ischemic attack, myocardial infarction, angina, smoking, alcohol, diabetes mellitus, cholesterol, anti-hypertensive drugs, and history of hormone replacement therapy (for women). Depression was assessed as a continuous variable by the Geriatric Depression Scale [35]. *APOE* genotyping was coded as either carriers or non-carriers of the <$>\raster="rg1"<$>4 allele. BMI was calculated as the ratio of weight (kilograms) to height (metres) squared. Smoking was defined as whether participants had ever regularly smoked or not. Alcohol was divided into categories determined by the number of drinks participants had on a daily basis (0, 1–2 or Over 2) within the last year. Diabetes Mellitus was defined as either having a previous diagnosis made or a Fasting Blood Glucose ≥7.0 mmol/L.

### Statistical Analyses

Preliminary analyses were carried out to ensure no violation of multicollinearity. Linear multiple regression analyses were used to assess the associations of PWV with the global cognitive measure and the cognitive domains. The continuous PWV measure was converted to a binary variable in order to compare subjects with PWV measurements in the top and bottom tertiles of the cohort. Analyses of covariance (ANCOVAs) were used to investigate the relationship between this binary variable and cognition. Both regression and ANCOVAs were carried out with two sets of covariates, the first model incorporating age, sex and years of education as covariates (Model 1) and the second model adding the additional covariates listed in the previous section (Model 2). To examine possible sex differences in these relationships, the analyses were repeated separately for male and female subsamples. The statistical significance of any differences between males and females in these results was determined by examining the interactions between sex and PWV in the regression and ANCOVA models. Bonferroni correction for multiple testing was used for the series of analyses investigating the relationships between PWV and the cognitive domain scores. Correction was set at (p<0.0125), calculated by dividing 0.05 by four dependent variables (cognitive domains).

## Results

### Descriptive statistics

Table 1 presents characteristics of the study sample. The mean age was 79.6±4.2 years, 53% of the sample was female and the mean duration of education was 11.8±3.6 years. Table 2 shows the mean and standard deviation of composite global cognition and the four cognitive domains.

**Table 1:** General Characteristics of those in the Study Sample.

	Males	Females	All
	n = 154	n = 165	n = 319
	Mean (SD)/%	Mean (SD)/%	Mean (SD)/%
Age (yrs)	79.7 (4.1)	79.5 (4.3)	79.6 (4.2)
Years of Education	12.7 (3.9)	11.2 (3.2)*	11.9 (3.6)
BMI (kg/m2)	27.2 (3.6)	26.2 (4.4)*	26.7 (4.1)
PWV (m/s)	11.6 (2.7)	10.8 (2.1)*	11.2 (2.4)
Systolic Blood Pressure	141.7 (18.5)	140.2 (19.9)	140.9 (19.3)
Pulse Rate	66.7 (11.4)	69.1 (9.8)*	68.0 (10.6)
Score on Geriatric Depression Scale	2.2 (2.1)	1.8 (1.7)*	2.0 (1.9)
Cerebrovascular Accident (CVA)	5.6%	0%*	2.6%
Transient Ischemic Attack (TIA)	5.0%	5.4%	5.2%
Acute Myocardial Infarction (MI)	16.8%	3.9%*	9.9%
Angina	16.8%	7.3%	11.7%
Currently taking anti–hypertensive medication	60.2%	60.3%	60.3%
Diabetes Mellitus	16.2%	6%*	10.8%
Fasting Blood Glucose			
>10 mmol/l	1.2%	0.5%*	0.8%
7.0–10.0 mmol/l	10.5%	2.5%*	6.2%
5.6–6.9 mmol/l	47.1%	42.2%*	44.5%
<5.6 mmol/l	41.3%	54.8%*	48.5%
Alcohol Consumption			
0 Drinks per day	6.7%	15.6%	11.5%
1 –2 Drinks per day	67.4%	78.0%*	73.1%
More than 2 drinks per day	25.8%	6.3%*	15.4%
Ever Smoked Tobacco Regularly	64.2%	38%*	50.3%
Total Cholesterol	4.3 (0.9)	4.9 (0.9)*	4.6 (1.0)
Apolipoprotein e4 allele	24.6%	22.9%	24.0%

* indicates a statistically significant difference between males and females (p<0.05).

**Table 2:** Mean and standard deviation of z-scores of global cognition and the four cognitive domains.

Cognition measures as z-scores	Males	Females	All
	n = 154	n = 165	n = 319
Processing Speed	0.12 (0.89)	0.12 (1.03)	0.12 (0.96)
Memory	−0.20 (0.91)	0.38 (0.93)*	0.11 (0.97)
Visuo-spatial	0.10 (1.00)	0.02 (1.03)	0.06 (1.01)
Executive Function	0.12 (0.97)	0.05 (1.00)	0.08 (0.99)
Composite Global Cognition	0.11 (0.93)	0.14 (1.01)	0.12 (0.97)

Cognition measures are presented as Z-scores based on the means and standard deviations of the whole Wave 2 sample. * indicates a statistically significant difference between males and females (p<0.05).

### Association between PWV and Composite Global Cognition

#### PWV as a continuous measure

A linear regression analysis, with composite global cognition as the dependent variable and PWV as the independent variable, showed no significant relationship between PWV and composite global cognition levels (B = −.02; t_318_ =  −.39; *p* = .70) for either model (results for Model 2 are presented). When the sexes were analysed separately, a significant association between PWV and composite global cognition was observed for men (B = −.18; t_153_ =  −2.06; *p* = .042) but not for women (B = .10; t_164_ =  1.30; *p* = .19), in both models. The correlation between PWV and cognition in men was −0.205, and in women it was −0.079. In an analysis using the whole sample, the interaction between sex and PWV was not found to be statistically significant for an alpha rate of.05, although p-values less than.10 for the interaction effect were found for both models (F (1, 318) = 2.85 p = .092 for Model 1, and (F (1, 318) = 3.11, p = .079 for Model 2). A stepwise approach did not change the results.

#### PWV as a categorical measure

Analysis of covariance was performed to assess differences in cognition between subjects with PWV measurements in the top and bottom tertiles of the cohort. The bottom tertile included subjects with PWV less than or equal to 9.8 m/s. The top tertile include subjects with PWV greater than or equal to 11.9 m/s. No significant relationship between PWV (categorical) and composite global cognition levels was found in the combined cohort under either Model 1 or Model 2 (F (1, 211) = 1.237; *p* = .267). However, when categorizing by sex, PWV was significantly associated with composite global cognition in men (F (1, 108) = 7.526; *p* = .007) but not in women (F (1, 102) = .762; *p* = .385). However, the interaction between sex and the PWV binary variable was not statistically significant for either of the models (*p*<.05).

### Association between PWV and Cognitive Domains

#### PWV as a continuous measure

No statistically significant associations between PWV (continuous) and any of the cognitive domains were found in Model 1 or Model 2. When the sexes were analysed separately, a significant association between PWV and memory was found in men (B = −.20; t_153_ =  −2.15; *p* = .034), but this did not reach significance after Bonferroni correction (adjusted alpha rate = .0125; See Table 3). A stepwise approach did not change the results.

**Table 3:** Association between pulse wave velocity (continuous) and cognitive domains.

	Male			Female			Total		
	n = 154			n = 165			n = 319		
Domain	β	t-score	Sig.	β	t-score	Sig.	β	t-score	Sig.
ProcessingSpeed	−0.117	1.198	0.233	0.044	0.481	0.631	−0.020	0.326	0.745
Memory	−0.203	2.147	0.034*	0.20	0.197	0.844	−0.082	1.424	0.156
Executive	−0.109	1.197	0.233	0.079	0.958	0.339	0.012	0.201	0.841
Visuospatial	−0.069	0.714	0.477	0.098	1.194	0.234	0.017	0.273	0.785

* p<0.05 analysis-wise, none was significant after Bonferroni correction (p<0.0125). Covariates: Age, Years of Education, BMI, Pulse Rate, Systolic BP, Cholesterol, Geriatric Depression Scale Score, ApoE genotype, daily alcohol intake, hormone replacement therapy, history of smoking, use of anti-hypertensive medication, history of cerebrovascular accident, myocardial infarction, transient ischaemic accident, angina, diabetes, and sex for whole sample analyses.

#### PWV as a categorical measure

An analysis of covariance, comparing the top and bottom tertiles of PWV, found no significant associations between PWV (categorical) and cognitive domains for either model (see Table 4). When the sexes were analysed separately, a significant association between PWV and memory was found in men (F (1, 108) = 8.190; *p* = .0096). No significant associations were found in women. The interaction between sex and the PWV binary variable was not statistically significant for either of the models.

**Table 4:** Association between pulse wave velocity as a categorical variable and cognitive domains.

	Male		Female		Total	
	n = 109		n = 103		n = 212	
	df = 108		df = 102		df = 211	
Domain	F Value	Sig.	F Value	Sig.	F Value	Sig.
Processing Speed	1.497	0.224	0.273	0.603	0.232	0.631
Memory	8.190	0.005#	2.144	0.147	1.051	0.306
Executive	1.802	0.183	0.00	0.985	0.492	0.484
Visuospatial	2.833	0.096	0.053	0.818	0.993	0.320

# significant after Bonferroni correction (p<0.0125). Covariates: Age, Years of Education, BMI, Pulse Rate, Systolic BP, Cholesterol, Geriatric Depression Scale Score, ApoE genotype, daily alcohol intake, hormone replacement therapy, history of smoking, use of anti-hypertensive medication, history of cerebrovascular accident, myocardial infarction, transient ischaemic accident, angina, diabetes, and sex for whole sample analyses.

## Discussion

This study examined the cross-sectional relationship between arterial stiffness, as measured by pulse wave velocity (PWV), and cognitive function within a non-demented community-dwelling elderly cohort. Results showed no significant association between arterial stiffness and global cognition. A trend was found when examining the interaction effects of sex and PWV in cognition. When stratifying the sample by sex, negative associations between PWV and composite global cognition were observed in men, but not in women. The same associations were identified, within men only, when comparing subjects with PWV measurements in the top and bottom tertiles of the cohort.

Similar results were displayed for the association between PWV and cognitive domains. Results showed no significant association between arterial stiffness and any of the four cognitive domains. However, when stratifying the sample by sex, negative associations between PWV (both continuous and categorical) and the memory domain were observed in men. The categorical association remained significant after Bonferroni correction.

Our results are in conflict with several other studies and this is likely due to our use of a more comprehensive array of covariates than other identified studies, and our incorporation of a Bonferroni correction for multiple testing, similarly not applied with any other cohorts. Our sample displayed some differences to previous studies. For example, our cohort is significantly older than comparable community based studies [7], [8], [11], [12], [13], [16], [17] but is overall a healthy cohort, most being of middle socioeconomic status. Differences in the relationship between arterial stiffness and cognitive function between men and women have been found previously. Waldstein, et al. (16) reported men as scoring significantly lower than women on tests of verbal memory at higher levels of Pulse Pressure, an alternative measure of arterial stiffness.

Furthermore, men in this cohort displayed significantly poorer outcomes as compared to women on several health measures, as well as a significantly higher mean PWV, higher proportion with MCI [36] and lower memory function. These findings, however, may not be specific to our cohort. It is well known that there are significant differences between the sexes in regards to vascular function and the timeline of vascular change during aging. Across the human lifespan, male risk for cardiovascular disease increases in a linear fashion, while females have a comparatively low CVD risk profile until menopause after which they gradually catch up with males [37]. Significant sex differences in cognitive function in the elderly have also been observed. A higher prevalence of mild cognitive impairment was reported for men in the Mayo Clinic Study of Aging and in our cohort [36], [38]. A possible hypothesis for this trend is that men experience cognitive decline earlier in life at a gradual pace, while women transition from normal cognition to dementia more abruptly [38].

Our study has major strengths including a relatively large sample size and the use of a comprehensive array of covariates and neuropsychological tests. Our stringent statistical criteria through use of Bonferroni correction for multiple analyses minimise type 1 errors. The major limitation of this study is its cross-sectional design.

In conclusion, our results only partially support the hypothesis of a negative association between PWV and cognition. An association between higher PWV and lower memory scores was only found in men. Our failure to find a significant relationship in the whole sample does not support the potential for the future use of PWV measurement within a clinical setting as an indicator for risk of cognitive decline. Further studies utilising comprehensive lists of covariates, rigorous statistical procedures with longitudinal designs are needed to clarify the relationship between increased arterial stiffness and decreased cognitive function.
